# Implementation of Mental Health Centres Pilots in Poland since 2018: A Chance to Move towards Community-Based Mental Health Services

**DOI:** 10.3390/ijerph19095774

**Published:** 2022-05-09

**Authors:** Anna Sagan, Iwona Kowalska-Bobko, Daria Biechowska, Maciej Rogala, Małgorzata Gałązka-Sobotka

**Affiliations:** 1 European Observatory on Health Systems and Policies, London School of Economics and Political Science, London WC1H 9SH, UK; 2 European Observatory on Health Systems and Policies, London School of Hygiene & Tropical Medicine, London WC1E 7HT, UK; 3 Institute of Public Health, Faculty of Health Sciences, Jagiellonian University Medical College, 31-066 Kraków, Poland; iw.kowalska@uj.edu.pl (I.K.-B.); maciej.rogala@uj.edu.pl (M.R.); 4 Department of Public Health, Institute of Psychiatry and Neurology, 02-957 Warszawa, Poland; dbiechowska@ipin.edu.pl; 5 Institute of Healthcare Management, Faculty of Economics and Management, Lazarski University, 02-662 Warszawa, Poland; m.galazka-sobotka@lazarski.edu.pl

**Keywords:** mental health, healthcare, coordination, integrated care, Poland

## Abstract

Provision of mental health care in Poland has long been characterised by an overreliance on psychiatric hospitals and the underdevelopment of community care. The introduction of the first National Mental Health Protection Programme for 2011–2015, with the explicit goal to base provision of mental care on the community mental health centres, failed to achieve any tangible results. The ensuing critique led to the launch of the second National Mental Health Protection Programme for 2017–2022 and the establishment, from mid-2018 onwards, of 41 (33 in operation) mental health centres across Poland. These will be piloted until the end of 2022 but have already shown positive results in terms of access to non-stationary care and a small fall in hospitalisations. They have also performed well during the COVID-19 pandemic, allowing for a quick reorganization of care and continued provision of mental health services. Some of the key innovations of the new model include the introduction of recovery assistants (a new profession) and mental health coordinators (a new role); liaison with social assistance services; and a shift to budget financing. The key obstacles to the national rollout of mental health centres are the low financing of mental health care in Poland, which is among the lowest in Europe, and acute workforce shortages.

## 1. Introduction

About 12% of the population in the WHO European Region suffers from mental disorders at any given time [1]. Inclusion of substance use disorders increases this share to 15%, while the inclusion of neurological disorders such as dementia raises it to 50%. According to the WHO, by 2030, depression will be the greatest contributor to the global burden of disease [2]. While there are no systematic epidemiological studies of mental disorders in the general population in Poland [3], a representative survey conducted in 2012 found that about 23% of the Polish population aged 18–64 suffered from mental disorders [4]. Of these, the most common were alcohol abuse (11.90%), specific phobias (4.3%) and depression (3.0%). The number of patients diagnosed with these disorders and receiving treatment increased steadily between 2014 and 2016 [3]. Death by suicide, which is one of the leading symptoms of mental health problems, remains much higher in Polish men (21 per 100,000 in 2018) compared to the EU average (17 per 100,000 in 2016) [5]. Mental and behavioural disorders account for the largest share (over 17%) of the benefits paid out by the Social Insurance Institution to persons with short- and long-term incapacity to work [6].

In much of Europe, mental health had long been one of the most neglected areas of public health, with systemic, organisational, legal, and social barriers contributing to the exclusion of people with mental health problems [7]. The need to develop community-based mental health care as an alternative to institutional care in mental health asylums has been widely recognized (e.g., [7,8]). Over the past two decades, many countries in Europe have managed to significantly reduce the number of psychiatric hospital beds, with the largest reductions observed in Cyprus and Ireland (over 70%), followed by Italy, Malta, Netherlands, Finland, Iceland, Norway, and the United Kingdom (over 40%) [9]. In comparison, the number of psychiatric hospital beds in Poland fell by only about 10% in this period.

The purpose of this perspective piece was to describe the introduction of a pilot of coordinated mental health care in mental health centres as a primary means for achieving a shift from asylum-based to community-based mental health provision in Poland. Section 2 describes the policy background, including earlier efforts to improve provision of mental health services. Section 3 summarises the content of the new policy and its early results. Finally, Section 4 offers conclusions and policy recommendations.

## 2. Policy Background

The first National Mental Health Protection Programme introduced in Poland was adopted in 2010 and was in place between 2011–2015 [10]. The goal of this programme, in line with the developments in other countries in Europe, was to shift provision of mental care from hospitals to the community by introducing a network of mental health centres as the core element of mental health service provision. These were first to be piloted at a smaller scale and the Minister of Health was charged with working out details of the pilot and ensuring its financing and implementation. The National Health Fund (NHF), the sole payer in the public health care system, was mandated with developing an appropriate financing model for the centres. Unfortunately, the Ministry did not fulfil its tasks and did not establish the principles for creating and financing the centres, precluding other actors, including the NHF, from implementing theirs. The inaction on the part of the Ministry was ascribed to the lack of financial resources to implement the programme but also to the lack of agreement among the key stakeholders about the details of the pilots and the overall model of psychiatric care that should be pursued in Poland [11]. Given the problems mentioned above, the assessment of the programme conducted by the National Audit Office in 2016 was unsurprisingly scathing [11]. According to the report, the number of fatal suicide attempts—one of the key indicators available for monitoring the implementation of the programme—increased by over 60% between 2011 and 2015.

Financing of mental care services remains extremely low, with psychiatric care and addiction treatment accounting for just over 3% of the NHF’s expenditure [12], which is among the lowest shares in Europe [13]. About 70% of these funds are allocated to residential care, mostly to dedicated psychiatric hospitals, where 11,000 or two-thirds of psychiatric beds are located (only 5450 beds are in general hospitals) [14], and which may contribute to social stigmatisation of psychiatric patients [15]. Only about 30% of public funds are allocated to non-residential care. Further, the majority of service providers (about two-thirds) are contracted to provide only one form of care (i.e., either outpatient, community, day, or emergency (hospital) care), which combined with poor cooperation among the various providers means that most patients do not have access to comprehensive and coordinated psychiatric care [16]. Many patients diagnosed with mental health disorders thus turn to primary health care (PHC) to seek help (about 875,000 out of 1.5 million patients in 2019) and many do not receive specialist care [17].

Although the number of psychiatric wards decreased slightly (by 4%) between 2010 and 2016 and the number of community care units increased, the percentage of patients with mental disorders using these community forms of treatment remained small—only 1.9% of patients used community mental care and 1.6% used day mental care [18]. Access to community care remains highly unequal, with only a third of the counties having a community (home) treatment team and a similar share having a day ward [13,16]. For example, in 2018, over 95% if patients with schizophrenia were treated in hospital settings and only 5% had access to a comprehensive care in both hospitals and other settings, including day and community care [19].

Analysis of treatment pathways between 2010 and 2016 further revealed that emergency wards were most often used by those patients who were also treated at outpatient mental health clinics and hospitalised in 24 h care wards. This may be a further indicator that the availability of outpatient services is not sufficient, and that the emergency wards were used to access needed care [18]. At the same time, better access to day care seems to prevent round-the-clock hospitalisation and visits to emergency wards. The lowest rates of hospitalisations in general psychiatric departments could be found in the counties in which all forms of mental health care were available [18].

It was not until after the scathing report by the National Audit Office was published [11] that the Minister of Health appointed (in April 2016) a dedicated team of experts charged with designing the details of the pilot of community mental health centres. In March 2017, the second National Mental Health Protection Programme for 2017–2022 [20] was introduced offering a new chance to accelerate transformation of mental health service provision. The programme defined a set of actions aimed at providing people with mental disorders with comprehensive, multifaceted, and universally accessible health and other care and support services to enable them to function in their familial and social environments—as before, this was to be achieved by implementing a network of mental health centres, with the structure of the centres remaining similar to the one proposed in the previous programme and in line with the general approach to organising community mental health services in Europe [21]. These goals were supported in the consecutive editions of the National Health Programme—for 2016–2020 [22] and for 2021–2025 [23]—which is one of the key strategic planning documents in public health in Poland.

## 3. Policy Content and Early Results

The piloting of mental health centres started in July 2018 [24]. The centres are meant to provide adult populations living in their catchment areas with tailored and comprehensive psychiatric assistance close to their place of residence. All centres should provide the following types of services: active long-term treatment and support for people with chronic mental health disorders; short-term assistance for people with episodic or recurrent disorders; ad hoc assistance for people with urgent problems; as well as consultations for other people requiring diagnostic services or advice [20]. Their catchment areas should cover between 50,000 and 200,000—and ideally 100,000–120,000—adult inhabitants and may comprise an area of a larger county or several smaller counties, a smaller city, or a district of a larger city, depending on the population density. The centres receive lump-sum financing (a form of global budgets), allowing them flexibility in spending and to tailor provision of services to local needs. The financing is calculated as a product of the number of inhabitants and a capitation rate, which is the same for all centres and is indexed annually to account for changes in prices.

**Figure 1:**
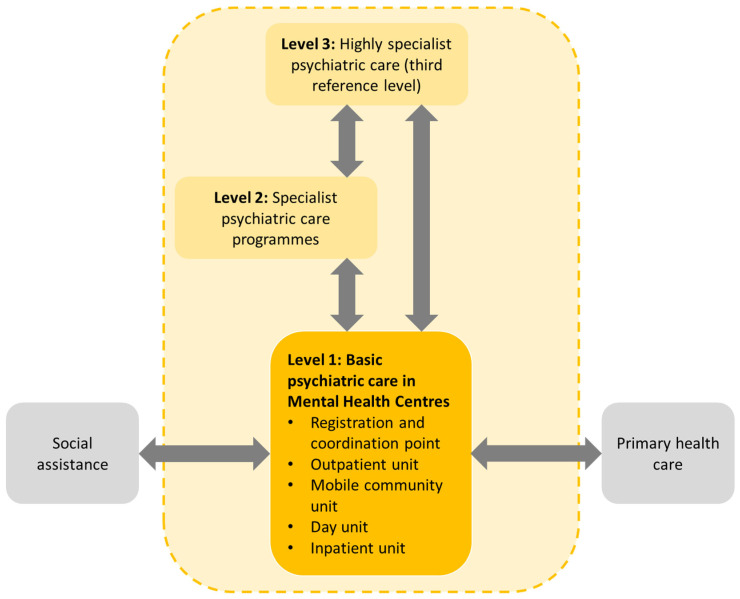
Organization of the mental health centre model of community mental care. Source: authors based on [28].

In terms of their organizational structure, the centres should comprise at least the following units (Figure 1): an outpatient unit or clinic providing medical and psychological advice, individual and group psychotherapeutic assistance, nursing services, and social interventions; a mobile community unit providing home visits, individual and group (incl. family) therapy, skills training, rehabilitation services, and general assistance to patients in building a social support network; a day unit providing day psychiatric hospitalisation to support provision of diagnostic, therapeutic or rehabilitation interventions; and a hospital unit, ideally located within a local general hospital rather than a specialist psychiatric hospital, providing round-the-clock hospital care for patients suffering from or at risk of severe disorders [20]. The centres may also sign agreements with providers of addiction treatment services. Depending on local needs, the centres may comprise additional specialized teams catering to the needs of selected groups of patients (e.g., psychogeriatric teams) or provide special services and other support mechanisms, such as crisis assistance, crisis housing, etc. To ensure ease of access, all these forms of assistance should be accessible within a single institution—the local mental health centre—and located within the designated catchment areas.

Each centre should have a registration and coordination point (approx. one point per 80,000 inhabitants) and be accessible for at least 10 h a day (8:00 a.m.–6:00 p.m.), Monday to Friday, that ensures quick (referral-free) access to a wide range of services and support from trained staff, including community therapists, psychiatric nurses, and psychologists.

A new healthcare profession—recovery assistant—has been introduced through the pilots, emulating similar solutions implemented in countries such as Germany, Switzerland, Great Britain, Sweden, Norway, and the Netherlands [25]. Recovery assistants are people who have experienced a mental problem themselves, and after appropriate training, provide peer support to people who are currently experiencing such problems. They work with therapeutic teams, providing a link between people seeking medical help and medical staff. A new role—mental care coordinator (case manager)—has also been introduced. Care coordinators are appointed by the head of the mental health centre from those among non-medical staff who have completed community therapy training. They ensure that a treatment and recovery plan is in place and is implemented and support patients and their relatives not only in the treatment process, but also in other areas, e.g., pertaining to their social life. So far, the education of existing health care professionals has not been adapted in line with the shift towards community mental care but the inclusion of a compulsory internship in community care in psychiatric specialist training is being considered.

Mental health centres should also cooperate with PHC, such as with primary care doctors consulting with the centres, as needed, to manage mild cases or for referring patients to the centres. The centres can also consult PHC doctors on specific cases [26]. They should also cooperate closely with the entities providing social support, social and professional activation, and other social assistance services. These close links with PHC and social services are meant to improve access to the centres and reduce stigmatisation of people with mental health problems [27].

The centres meet regularly (once a month) to exchange their experiences and learn from each other.

**Figure 2:**
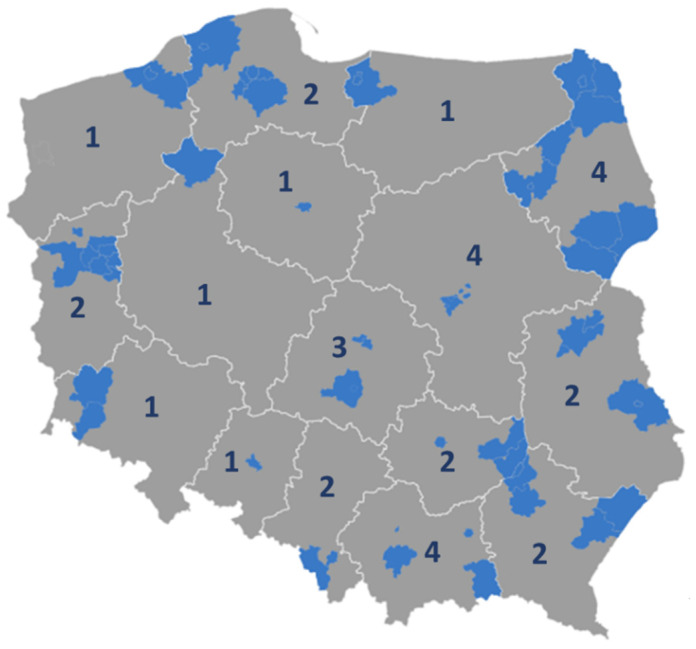
Number and location of mental health centres at the end of December 2021. Source: authors based on [30]. Notes: grey areas = regions; blue areas = catchment areas of mental health centres (this could be an area of one or more counties, or of a municipality or a smaller city, depending on the population density). The numbers represent the number of mental health centres in each region.

At the end of 2021, there were 41 mental health centres across the 16 regions of Poland, out of which 33 were in operation, covering 3.8 million people or 12% of the adult (18+) population (Figure 2) [29]. This reflects slow progress given that the reform assumed that 250–300 centres would be created by the end of 2027. This can be partly explained by the rigid inclusion criteria, precluding some of the interested entities from participation. Further, not all elements of the new model have been implemented. For example, in about half of the centres, lump-sum financing accounted for less than 50% of contracts, and care coordination and recovery plans were also only partially implemented [13].

At the end of 2020, the Ministry of Health and the Piloting Office of the National Mental Health Protection Programme published an assessment of the pilot in 27 centres between 2018 and 2019 [13]. The report compared two types of centres: in 17 centres, lump-sum financing constituted over 50% of the value of the contracts (or the total value of the contracts was less than PLN 10 million)—these centres usually operated out of psychiatric wards of general hospitals (Group 1); in the remaining 10 centres, lump-sum financing accounted for less than 50% of the contracts and they typically operated out of psychiatric hospitals (Group 2). The control group comprised all adult populations not covered by the pilot. Despite its slow and partial implementation, positive effects of the pilot have been determined, particularly in the centres belonging to Group 1 (see Table 1).

Between 2018 and 2019, access to mental health services in counties participating in the pilot improved while differences in access between these counties became smaller. For example, the difference between the number of treated patients between the countries with the highest and lowest numbers of treated patients per 100,000 inhabitants decreased by 10.5% and the difference in the number of patients receiving ambulatory and community care between the counties with the highest and lowest rates of access to such care decreased by 11.2%, while at the same, the average and median values for these two indicators increased across the participating countries [13]. Improved access to care could be linked to the increased spending in the centres which rose by 125% per patient between 2018 and 2019 [31]. The centres are also thought to have contributed to reducing access barriers and improving coordination of care. This was ascribed to provision of services in the registration and coordination points and the introduction of care coordination mechanisms, such as the new role of care coordinator, but care coordination and quality have not been comprehensively studied. Further, there were essentially no queues and almost no complaints to the Patient Rights Ombudsman at the centres compared to the control group [13]. In contrast, access to psychologists and psychotherapists outside of the pilots is only available upon a referral (although psychiatrists can be seen without a referral) and waiting times for consultations can be long—about 50 days on average according to 2019 data [32].

In terms of differences between different centres, centres in Group 1 noted an increase in the shares of their populations receiving mental health care: there was a significant increase in the provision of non-stationary care, with the number of outpatient and community services increasing by almost 7%; the number of psychological counselling and therapy services by 26%; and the number of inhabitants covered by community care by 27%. At the same time, hospitalisations have fallen slightly by just over 3% in the centres in Group 1. Centres in Group 2 have seen much smaller improvements, often smaller than in the control group. This has been attributed to the ‘institutional culture’ dominating in large psychiatric facilities where Group 2 centres are located; a smaller share of lump-sum financing compared to Group 1; as well as the lack of organisational and financial separation of the centres from the structures of the hospital and, related to that, the weak position of the centres’ managers, among other factors [13].

The new model also seems to have performed well during the COVID-19 pandemic [28,33]. While many psychiatric treatment wards were closed in the initial stages of the pandemic, mental health centres—thanks to their organisational and financial flexibility—managed to quickly adapt their operations and switch to remote provision.

## 4. Discussion

Early results of mental health centre pilots appear to be promising and they have been perceived positively by the regional medical consultants [16]. More information, including on care coordination and quality of care as mentioned above and on health outcomes, are needed to provide a more comprehensive assessment of the new model and inform any adaptations.

What is worrying is that many centres that have been created so far operate out of psychiatric hospitals with strong ‘institutional culture’. Progress towards community care may be slower in such centres and their location may not be conducive to reducing the social stigmatisation of mental health patients. The report of the Piloting Office recommended to extend the pilot to other entities, such as those participating in the EU-funded project “Deinstitutionalization of services provided to people with mental disorders and diseases” [28]. This project, in operation since 2015, comprises many entities that do not provide hospital services, including private entities and other non-governmental organisations, and has also been assessed positively in terms of improving access to mental health services in the community [34,35]. Inclusion of these entities in the pilot can offer potential for learning and for transfer of best practices to the centres and can help reinforce shifting mental care to the community. From 2022, entities without their own psychiatric ward have been permitted to apply for inclusion in the pilot—this should also help move mental care to the community and increase take up of the new model across Poland [36]. Based on the new applications from early 2022, the number of centres included in the pilot is expected to increase to about 80 in the first half of the year, reaching about 30% of the adult population [37].

Yet, there are many threats that can undermine the national rollout of the pilot. The key problem is the extremely limited public financing of mental health services. While the EU funds may provide the needed financial means to support the deinstitutionalisation of mental care, the level of national public financing is likely too low to ensure adequate provision in the long term. However, increasing public spending in this area may be difficult to achieve given other competing priorities and the relatively low interest of both central and local public authorities in addressing mental health problems (although this may also be explained by the limited local budgets) [16]. Ambivalent attitudes towards the reform are particularly notable in regions that are homes to large psychiatric hospitals.

Acute shortages of health workers constitute another major threat. In 2019, there were 10.2 psychiatrists per 100,000 inhabitants in Poland—much lower than the number recommended by the National Consultant in Psychiatrics (20 per 100,000; [17]), which is in line with the EU/EEA average [38]. However, mental health centres’ more flexible organisational structures which allow them to employ other mental care professionals other than psychiatrists may to some extent help mitigate the problem of staff shortages.

Initial plans assumed that the piloting phase would last three years, but due to the outbreak of the COVID-19 pandemic, this was extended until the end of 2022. In mid-2021, when the pilot was originally meant to end, the team of experts charged with the implementation of the pilot of community mental health centres also ceased to work and their operation was not extended. Instead, the Ministry of Health established a new team charged with the continuation of the reform and development of a new Mental Health Strategy for 2022–2027. Some health analysts have been concerned by the fact than none of the original experts were involved, especially since the results of the pilot have been generally perceived as promising. At the same time, the key strategic documents in the health sector, the National Transformation Plan for 2022–2026 published in late 2021 [39] and the framework document “Healthy future. Strategic framework for the development of the health care system for the years 2021–2027, with a perspective until 2030” [14] along with the Transformation Plan, postulate the development of community mental health care, including investments in human resources and infrastructure, and explicitly foresee further development of mental health centres over 2022–2027. While it remains unclear whether any additional entities will be included in the pilot in 2022, these strategic postulates seem to bode well for its national rollout when it comes to an end at the end of 2022.

## 5. Conclusions

Mental health centres offer a breakthrough in achieving a shift to community-based mental care in Poland. They provide local populations with an easily accessible entry point to comprehensive and coordinated mental and related services close to their place of residence and without a referral. The pilot covers 12% of the adult population and its early results have been promising: it has led to improved access to non-stationary care as well as a small fall in hospitalisations. However, its further implementation may be undermined by the acute health workforce shortages and low financing of mental health services in Poland and there is some uncertainty about the future direction of the reform. Given the increasing burden of mental health problems, intensified by the COVID-19 pandemic, it is now a good time to push for more investment and improving mental health services.

## Figures and Tables

**Table 1:** Changes in access to mental health services within and outside of the pilot, % age change between 2018 and 2019.

	Group 1 (Hospital Wards); 1.45 Million People	Group 2 (Psychiatric Hospitals); 1.43 Million People	Control Group; 28.56 Million People
Outpatient and community care (number of consultations, sessions, and visits per 100,000 inhabitants)	+6.7% (11.6% *)	+0.0% (+0.1% *)	+2% (+2.0% *)
Psychological counselling and psychotherapy * (number of consultations, psychotherapy sessions, group sessions, community therapist visits per 100,000 inhabitants)	+25.6%	+8.8%	+9.9%
Number of patients receiving community care per 100,000 inhabitants	+26.9%	−0.5%	+4.6%
Hospitalisations (number of bed-days per 100,000 inhabitants)	−3.3%	−1.8%	−0.5%

* Including services in the registration and coordination points. Source: authors based on [13].

## Data Availability

Not applicable.
